# Occupational Exposure to Diesel Motor Exhaust and Lung Cancer: A Dose-Response Relationship Hidden by Asbestos Exposure Adjustment? The ICARE Study

**DOI:** 10.1155/2015/879302

**Published:** 2015-09-03

**Authors:** Mireille Matrat, Florence Guida, Sylvie Cénée, Joelle Févotte, Matthieu Carton, Diane Cyr, Gwenn Menvielle, Sophie Paget-Bailly, Loredana Radoï, Annie Schmaus, Simona Bara, Michel Velten, Danièle Luce, Isabelle Stücker, The Icare Study Group

**Affiliations:** ^1^INSERM, Centre de Recherche en Epidémiologie et Santé des Populations (CESP), U1018, Equipe Epidémiologie des Cancers, Gènes et Environnement, Bâtiment 15/16, 16 Avenue Paul Vaillant Couturier, 94807 Villejuif, France; ^2^Faculté de Médecine, Université Paris Est Créteil, 8 rue du Général Sarrail, 94010 Créteil Cedex, France; ^3^Centre Hospitalier Intercommunal, Service de Pneumologie et Pathologie Professionnelle, 40 Avenue de Verdun, 94010 Créteil, France; ^4^Unité Mixte de Recherche Épidémiologique et de Surveillance Transport Travail Environnement (UMRESTTE), Université Claude Bernard Lyon 1, 69373 Lyon, France; ^5^INSERM, Centre de Recherche en Epidémiologie et Santé des Populations (CESP), Unité UMS 011 Equipe Cohortes Épidémiologiques en Population, Bâtiment 15/16, 16 Avenue Paul Vaillant Couturier, 94807 Villejuif, France; ^6^Université de Versailles Saint-Quentin, UMS011, Equipe Cohortes Épidémiologiques en Population, Bâtiment 15/16, 16 Avenue Paul Vaillant Couturier, 94807 Villejuif, France; ^7^INSERM, UMR_S 1136, Institut Pierre Louis d'Epidémiologie et de Santé Publique, 56 Boulevard Vincent Auriol, CS81393, 75646 Paris Cedex 13, France; ^8^UPMC Université de la Sorbonne, Université Paris 06, UMRS 1136, Institut Pierre Louis d'Epidémiologie et de Santé Publique, 56 Boulevard Vincent Auriol, CS81393, 75646 Paris Cedex 13, France; ^9^INSERM, Centre de Recherche en Epidémiologie et Santé des Populations (CESP), U1018, Equipe Epidémiologie des Déterminants Sociaux et Professionnels de la Santé, Bâtiment 15/16, 16 Avenue Paul Vaillant Couturier, 94807 Villejuif, France; ^10^Faculté de Chirurgie Dentaire, Université Paris Descartes, 1 rue Maurice Arnoux, 92120 Montrouge, France; ^11^Registre des Cancers de la Manche, Centre Hospitalier Public du Cotentin, 46 rue Val de Saire, 50102 Cherbourg, France; ^12^Registre des Cancers du Bas-Rhin, Département d'Épidémiologie et de Santé Publique, Faculté de Médecine, Université de Strasbourg, 4 rue Kirschleger, 67085 Strasbourg Cedex, France; ^13^INSERM U1085, IRSET, Campus de Fouillole, BP145, 97154 Pointe à Pitre, Guadeloupe; ^14^Université de Rennes 1, Campus de Fouillole, BP145, 97154 Pointe à Pitre, Guadeloupe

## Abstract

*Background*. In a French large population-based case-control study we investigated the dose-response relationship between lung cancer and occupational exposure to diesel motor exhaust (DME), taking into account asbestos exposure. *Methods*. Exposure to DME was assessed by questionnaire. Asbestos was taken into account through a global indicator of exposure to occupational carcinogens or by a specific JEM. *Results*. We found a crude dose response relationship with most of the indicators of DME exposure, including with the cumulative exposure index. All results were affected by adjustment for asbestos exposure. The dose response relationships between DME and lung cancer were observed among subjects never exposed to asbestos. *Conclusions*. Exposure to DME and to asbestos is frequently found among the same subjects, which may explain why dose-response relationships in previous studies that adjusted for asbestos exposure were inconsistent.

## 1. Introduction

Lung cancer is the leading type of occupational cancer, with 13% to 30% of the cases attributable to occupational exposures [1, 2]. Numerous lung carcinogenic hazards were/are present in the work place, in particular asbestos, a lung carcinogen very frequently found in numerous occupational settings such as construction, transport, isolation, and maintenance. In France, asbestos was banned by law in 1997. Nurminen and Karjalainen [3] showed in a large review dedicated to occupational attributable fractions that 14% of lung cancer cases were due to asbestos, 4.5% to radon, 2.7% to crystalline silica, and 2.5% to diesel motor exhaust (DME); figures close to those are found recently by De Matteis et al. [4].

In June 2012, the International Agency for Research on Cancer evaluated the carcinogenicity of diesel engine exhausts and classified these fumes as “carcinogenic to humans” (Group 1) [5]. This upgrading was achieved with the results of major cohorts which demonstrated dose response relationship between DME exposure and lung cancer in sectors where coexposure was not the main concern (i.e., underground mines). This evaluation, however, is discussed by some authors who question the validity of the results of cohort studies among miners [6, 7]. Other studies have also demonstrated an increase in lung cancer risk, although no consistent dose-response relationship was observed while considering occupational exposure to specific lung carcinogens such as asbestos or silica [8–14]. On the other hand, studies that were adjusted for broad groups of carcinogens (i.e., List A jobs or high risk jobs for lung cancer) did find a significant trend between level/duration of DME exposure and lung cancer [15, 16].

From a large population-based case-control study conducted in France in the 2000s, we investigated the relationship between lung cancer and occupational exposure to DME while considering the smoking habits and occupational exposure to asbestos.

We considered first list A as a proxy of asbestos exposure and then used a specific asbestos job exposure matrix to examine the influence of asbestos assessments on the dose-response relationship.

## 2. Material and Methods 

### 2.1. Study Design

The ICARE study is a large multicentre population-based case-control study conducted in France between 2001 and 2007 that has previously been described in detail [17]. Briefly, all lung and upper aerodigestive tract (UADT) cancer patients who were between 18 and 75 years of age and who were identified during the study period in each cancer registry were eligible for the study. The industrial activities in the* départements* (area of residence) with registry are representative for industrial activities in France. The cases were all histologically confirmed as primary lung cancer (C33-C34 ICD-O), including all histological types. Of the 3865 eligible men cases identified, 403 could not be located, 653 died before any contact could be made, and 197 could not be contacted because of their health status. Accordingly, 2612 patients were asked to participate, and 336 refused, which led to a refusal rate of 12.9% (comparable in men and women).

Population controls were randomly selected by a polling institute from the same* départements* as the cases through incidence-density sampling. The controls were frequency-matched to the cases by age (<50 years, 50–60, 60–70, and over 70), gender, and* département*. Additional stratification was performed to achieve a distribution by socioeconomic status among the controls comparable to that of the general population in each* département.* Among the 3618 eligible men controls, 208 could not be contacted, and 4 died before any contact could be made. Accordingly, 3406 were asked to participate and 626 refused (refusal rate: 19%).

Overall, 6481 subjects have been included in the study: 2926 cases and 3555 controls. We restricted the analysis to men (78% of our population, *n* = 2264 cases and 2780 controls) because only 104 women declared an exposure to DME.

The study was approved by the Institutional Review Board of the French National Institute of Health and Medical Research (IRB-Inserm, number 01-036).

### 2.2. Data Collection

The interviews were conducted in person by trained interviewers using standardised questionnaires. Information was collected on demographics, residential history, education, occupation, lifelong cigarette smoking, and alcohol consumption. If the subject was unable to answer the complete questionnaire, or if a relative answered for the subject (a spouse or a child), a summary version of the questionnaire was used that primarily included information on lifetime cigarette smoking and lifelong occupational history but not education level. This short version applied to 5% of the respondents (*n* = 201/2264 cases and 60/2780 controls).

The lifelong occupational history included the beginning and ending date of each job, the industry, a description of the tasks carried out in the job, the type of machines used, and the exposure to DME. Twenty supplementary questionnaires completed the interview for certain tasks or job titles including jobs in mines/quarries or in vehicle repair. The occupations and branches of industry were coded blindly with respect to case or control status by trained coders according to the* International Standard Classification of Occupations *(ISCO) of the International Labour Organisation, 1968 revision [18], and according to the French Nomenclature of Activities (*Nomenclature d'activités Françaises*: NAF) of the National Institute for Statistics and Economic Studies (INSEE), 1999 edition [19], respectively.

Lists of occupations or industries associated with lung cancer either conclusively (List A) or with suspicion (List B) were first established by Ahrens and Merletti [20] according to the evaluation of carcinogenic risk conducted by IARC. These lists have been repeatedly updated and extensively used worldwide as a standardized tool to quantify the burden of occupational lung cancer. We applied List A to our data using the latest update created by Mirabelli et al. [21] and detailed by Consonni et al. [22].

### 2.3. Assessment of DME Exposure

Assessment of DME exposure was done by job period with the single question of the general questionnaire “During this job period were you exposed to DME?” allowing us to assess a probability of exposure (*P*_*Q*_) to DME in three classes (yes/no/do not know). In addition, for those people who answered no or do not know to this question, but who had declared that they were working in mines or quarries close to a diesel motor or in a garage involved in truck repairing, we created a fourth class of probability defined as possibly exposed (*P*_*Q*_ became then 0 for no exposure, 1 for possible exposure, 2 for certain exposure, and missing data for “don't known”). We created the variable “maximal exposure probability for the lifelong occupational history” to classify the study population. If someone did not know if he had been exposed to DME and had no other job with a certain exposure to DME, the maximal exposure probability was considered as a missing data. However, for the calculation of exposure to DME duration for the lifelong occupational history, each job with an exposure probability to DME was considered, even if the maximal exposure to DME is missing.

### 2.4. Assessment of Asbestos Exposure

Asbestos exposure was assessed using an asbestos specific JEM [23, 24]. For each job *i*, according to the combination of ISCO and NAF codes, the asbestos-JEM assigned a probability of exposure (*P*_*i*_), a frequency (*F*_*si*_), and an intensity (*I*_*si*_) of exposure related to specific tasks and a frequency (*F*_*ai*_) and an intensity (*I*_*ai*_) of exposure related to work environment contamination. The probability of exposure was expressed with the percentage of workers exposed in the considered combination ISCO × NAF in 5 classes: none or less than 1% of workers exposed in the considered job code; <5%; 5–30%; 30–70% and >70%. The frequency of exposure corresponded to the percentage of working time during which a subject of this combination would be exposed in 4 classes (<5%, 5–30%; 30–70%; ≥70%). The intensity was given in fibers/mL in 5 classes (<0.01; 0.01–0.1; 0.1–1; 1–10; >10).

### 2.5. Assessment of Silica Exposure

Assessment of silica exposure had been done in a previous work, using a specific silica JEM [25]. Similarly to the asbestos JEM, for each job *i*, according to the combination of ISCO and NAF codes, the silica-JEM assigned a probability of exposure (*P*_*i*_), a frequency (*F*_*i*_), and an intensity (*I*_*i*_). The probability of exposure was expressed with the percentage of workers exposed in the considered combination ISCO × NAF in 12 classes: none or less than 1% of workers exposed in the considered job code; <5%; ]5–15%]; ]15–25%]; …; 85–95%; >95%. The frequency of exposure corresponded to the percentage of working time during which a subject of this combination would be exposed in 12 classes (0; ]1–5%], ]5–15%]; …, ≥95%). The intensity was given in mg/m^3^ in 5 classes 0; ]0.02–0.1]; ]0.1–0.5]; ]0.5–1]; >1.

### 2.6. Individual Assessment of Exposure

Each subject's exposure to DME was then summarised by the following exposure indices:

(i) the maximum probability of exposure based on questionnaires (*P*_*Q*_),
(ii) the duration of exposure corresponding to the sum of the duration of exposed job periods, whatever the probability of exposure to DME is,
(iii) the cumulative exposure index (CEI) corresponding to the product of the classes attributed to the exposure estimated by the questionnaire of each job period (*P*_*Q*_) with the duration of employment of the period, summed over all job periods of a subject. This calculation excluded the job periods for which the subjects did not know how to answer. The CEI was categorised into 3 classes (not exposed, low, high) according to the 50th percentile among controls corresponding to the following cut-off points 0 and 31 for the CEI calculated from the questionnaire's assessment.

Since asbestos exposure was assessed for adjustment purpose only, we solely calculated an asbestos cumulative exposure index as follows: CEI_asbestos_ = Σ*D*_*i*_ × *P*_*i*_ × [(*F*_*si*_ × *I*_*si*_)+(*F*_*ai*_ × *I*_*ai*_)], where *D*_*i*_ is the duration of job *i*. We assessed two asbestos cumulative exposure indices defined as sensitive for one and specific for the other according to the probability of asbestos exposure taken into account to calculate the cumulative index. The sensitive assessment considered all jobs with a proportion of exposed workers above 1% (*P*_*i*_ from 1% to >70%). The more specific definition did not consider the jobs with a proportion of exposed workers equal to or less than 30% (*P*_*i*_ > 30%). In practice, to compute the CEI we assigned a value to the probability corresponding to the middle of the range of the class, that is, for the sensitive definition: nonexposed = 0; <5% = 0.025; 5–30% = 0.175; 30–70% = 0.5; >70% = 0.85. The specific definition assigned a value equal to 0 for all jobs with a probability ≤30% and the same values as in the sensitive assessment for the other classes. Similarly the values assigned to the intensity and the frequency of exposure corresponded to the middle of the range of the class. In addition, when the exposure was related to an indirect exposure instead of a specific task done by the worker himself, the values were divided by 2 [24]. The CEI was then categorized into four classes according to the distribution among controls.

The cumulative index for silica exposure was calculated similarly (i.e., silica = Σ*D*_*i*_ × *P*_*i*_ × *F*_*i*_ × *I*_*i*_) and then categorized into four classes according to the distribution among controls.

### 2.7. Statistical Analyses

Unconditional logistic regression was used to estimate odds ratios (OR) and 95% confidence intervals (95% CI) for association between lung cancer and different diesel exhaust exposure indices.

Lifelong cigarette smoking was captured by the cumulative smoking index (CSI) (CSI = (1 − 0.5^dur^*∗*^/*t*^)(0.5^tsc^*∗*^/*t*^)ln(int + 1), where *t* is the half-life parameter, tsc^*∗*^ = max(tsc − *d*, 0), and dur^*∗*^ = max(dur + tsc − *d*, 0) − tsc^*∗*^, *d* is a lag time parameter, and int is the average number of cigarettes smoked per day), which takes into account the total duration of smoking, time since cessation (TSC), and average number of cigarettes smoked per day [26]. In our data, it varied linearly with lung cancer risk and was used as a continuous variable for adjustment. A nonsmoker was considered having smoked less than 100 cigarettes in his lifetime. The CSI of never smokers is null. For effect of modification purpose, we considered smoking status in 3 classes: nonsmoker, ex-smoker, and current smoker.

Models were adjusted for age at interview (<50 years, [50-60[, [60-70[, and ≥70), the* département*, the CSI and the total number of working periods (<3 jobs, ]3-4], ]4-6] and >6 jobs, according to the quartiles in the controls) and either employment in a “List A” job (yes, if the subject had held at least one job included in this list /no, if not), asbestos CEI in four classes with cut-off point at 0.27 and 14 or 2.06 and 28 according to the sensitive or specific asbestos assessment, respectively, or silica CEI, in 4 classes with cut-off point at 0.048 and 0.495. Adjustment for List A or for asbestos or silica exposure was never run in the same models.

Modification effect between asbestos exposure or smoking status and DME in the risk of lung cancer has been tested with logistic regression by likelihood ratio method.

The analysis was first conducted on the entire male population and was subsequently restricted to men who were never exposed to asbestos.

Population attributable fraction (PAF) associated with DME exposure and the corresponding 95% CI were estimated, using the proportion of people definitely exposed to DME among asbestos never exposed subgroup and the OR adjusted for age, department, lifelong cigarette smoking, and number of jobs [27, 28].

Statistical analyses were performed using SAS software (SAS Institute Inc., Cary, NC, USA).

## 3. Results

A total of 5044 men (2264 cases and 2780 controls) who reported their occupational histories were included in this analysis. Their main sociodemographic characteristics are presented in Table 1.

The mean age at diagnosis was 60 for the cases and 58 for the controls (*p* < 10^−4^). The cases had a lower educational level than the controls. We observed a clear increase in the risk of lung cancer with the cumulative smoking level assessed by the CSI (*p* trend <10^−4^). The two main histological types were squamous cell carcinoma (*n* = 803, 35.2%) and adenocarcinoma (*n* = 794, 34.8%).

Having held at least one job period with a potential exposure to a carcinogenic agent (i.e., List A = yes) is associated with a higher risk of lung cancer (OR = 1.8 (95% CI 1.5–2.1)) (Table 2).

In addition, results demonstrate a clear dose effect relationship with asbestos exposure, whatever the definition of asbestos assessment, sensitive or specific. In both cases, the *p* value of the trend is highly significant, and the increased risk is manifest from the lowest cumulative exposure class to the highest (OR = 1.5 (95% CI 1.2–1.7) or OR = 1.2 (95% CI 1.0–1.5) for the sensitive or specific asbestos assessment to 2.3 (95% CI 1.7–3.0) or 1.7 (95% CI 1.2–2.3), resp.).

In this study, the occupational exposure to DME concerned the period of time between 1944 and 2006, when data collection ended.  Table 3 presents the results of the association between DME exposure and lung cancer assessed with the maximum probability of exposure from questionnaire (*P*_*Q*_). Thirty-five percent of the cases and 29% of the controls had declared at least one job period with a DME exposure. We observed an increased risk of lung cancer for the subjects who declared to be exposed to DME (OR = 1.3 (95% CI 1.1–1.6)). No relation with duration of exposure was observed. The more the cumulative exposure index increases, the more the risk of lung cancer increases (OR = 1.4 (95% CI 1.1–1.6) for the highest IEC). These results were first adjusted for having held at least one job belonging to List A. No effect modification between asbestos exposure assessed in a sensitive way and DME in the risk of lung cancer could be detected (*p* = 0.66). As can be seen in Table 3, the adjustment for asbestos cumulative exposure slightly reduced the associations, especially when asbestos was assessed in a sensitive way (OR_3_). We also stratified the association between DME and lung cancer by smoking status. Results were rather similar among nonsmokers (OR_PQdefinite  exposure_^1^ = 1.37 (95% CI 0.67–2.7)), ex-smokers (OR_PQdefinite  exposure_^1^ = 1.27 (95% CI 0.98–1.64)), and among current smokers (OR_PQdefinite  exposure_^1^ = 1.46 (95% CI 1.1–1.9)), and no modification effect could be detected (*p* = 0.35) (data not shown).

We repeated the analysis among subjects never exposed to asbestos, assessed in a sensitive way (CEI_asbestos_ equals 0) (Table 4). This subgroup consisted of 638 cases and 1147 controls, corresponding to 511 jobs exposed to DME. The description of the main tasks of the job (free text) reported that it was often jobs with many hours of driving, for example, 16% of transport equipment operators, 11% of farmers, 6% of agriculture and animal husbandry workers, 6% technical salesmen, commercial travellers, and manufacturers' agents, 5% salesmen, shop assistants, and related workers). Interestingly, all associations studied (maximum of probability *P*_*Q*_, duration of exposure (≤ or >15 years), and cumulative exposure index) showed significant dose-response relationships. The additional adjustments with List A or silica IEC showed an increased significant risk of lung cancer.

We estimated a PAF of lung cancer to DME of 7.0% (95% CI 1.3–12.4) based on an OR of 1.46 (corresponding to the association obtained among subjects never exposed to asbestos, adjusted for age, department, lifelong cigarette smoking, and number of jobs) and a prevalence of exposure of 17% among controls and 22% among cases. Considering the whole population, with an OR adjusted for asbestos exposure (specific definition) of 1.26 (95% CI 1.05–1.51) we estimated a PAF of 7.2% (95% CI 1.8–12.3).

## 4. Discussion

With this study we were able to find a clear dose-response relationship between cumulative DME exposure and lung cancer risk among subjects who were never exposed to asbestos. Because in our population some occupations were exposed to both DME and asbestos, adjusting for asbestos decreased the associations highlighted, predominantly in occupations with a high exposure to DME, particularly when asbestos exposure was assessed in a sensitive way.

ICARE is a large population-based case-control study that was designed to investigate the role of occupational exposure in the risk of lung cancer. Cases and controls were stratified by sex and age using a single control group for both types of cancer (lung and upper aerodigestive tract cancers). Collaboration with the French network of cancer registries allowed us to recruit lung cancer cases in almost all of the healthcare establishments in the* départements *covered by the registries. Overall, less than 20% of subjects refused to participate in the study, and this percentage was similar in cases and controls. Furthermore, the 10* départements* included in the study covered a large fraction of the French population (13%) giving a broad view of the different situations of DME exposure. Therefore, our results may be extrapolated to the overall French population and to similar industrialised Western countries. In this context, we found that 7% of lung cancer cases in men could be attributed to DME, thereby accounting for approximately 1975 persons each year in France. This is almost three times higher than what was observed in the Nurminen and Karjalainen study, where there was an attributable fraction of 2.5% [3].

The retrospective assessment of exposure in case-control studies is always a matter of debate. Our questionnaire was specifically designed to assess occupational exposure to a large variety of carcinogens present in the workplace. Diesel exposure is relatively easy to assess, as it comes from classical and typical machines that operate with diesel engine. In addition, no specific JEM had been developed at the Department of Occupational Health of the “Institut de Veille Sanitaire” (National Institute for Public Health Surveillance). In our population, 29% of the controls reported at least one job with DME exposure, a number similar to that reported in an Italian population and slightly more than the frequency observed in Canada (14%) [11, 13]. However, diesel engines are more frequent in Europe than in North America. Because this exposure assessment was self-reported by the subjects, it is not possible to exclude completely differential misclassification bias in our results. Nevertheless, in order to minimize it, we presented the study to the subjects as a study aimed at investigating the relation between environmental exposures and health. In addition, data collection was set up between 2002 and 2007, before classification of DME in group 1 of carcinogens for lung. It is also required to consider residual confounding by smoking to explain these results. The CSI is a smoking index that takes into account the 3 main smoking parameters in the risk of lung cancer (intensity, duration, and time since cessation). This index varies linearly with the risk allowing optimal adjustment. The very low number of cases of never smokers exposed to DME (*N* = 14) makes unlikely a noncontrolled effect for passive smoking in our results.

Our results are in accordance with the two major meta-analyses [9, 29] published about a decade ago and more recently with the large pooled analysis from Olsson et al. [15].

Our results do show a clear impact of the adjustment for previous asbestos exposure on the crude findings. Asbestos exposure assessment was made in two ways (i) using List A as a proxy of occupational exposures including asbestos and (ii) using an asbestos specific JEM developed at the National Institute for Health Surveillance and already applied in one mesothelioma case-control study [24]. Then, to explore to what extent asbestos could impact the crude relation initially obtained, we considered a sensitive definition to calculate the cumulative index (taking into consideration all jobs titles with a proportion of exposed workers different from zero) and a more specific one for which the jobs with a proportion of exposed workers less than 30% were not taken into account in the calculation of cumulative index. First of all, as can be seen in Table 3, the two definitions of asbestos, sensitive and specific, are both related to an increase risk of lung cancer in a dose dependant way, although the more specific definition is slightly less steep. We also note that subjects with a low cumulative index were in both cases (sensitive or specific) at significant increase risk of lung cancer.

The impact of asbestos exposure varies according to the asbestos definition. The very minor changes between crude and adjusted results with List A indicate that this list underestimates asbestos exposure and is not a way to accurately take asbestos into account. Although List A is a very useful tool to consider carcinogenic occupational exposures in a whole in investigations not specifically targeted to an occupational agent, it seems that this list is too global to take into account as precisely as possible a particular agent, such as asbestos, very frequently found in different workplaces in particular relevant periods for our cases and controls who were mainly active before the asbestos ban in France.

Our results with asbestos exposure adjustment show that workers exposed to diesel fumes may also have been exposed to asbestos. These jobs belong to the two following ISCO-68 groups: 8-4 (group including assembler fitters, machine installers, and precision mechanics and a group consisting notably of agricultural, automobile, or truck mechanics, and in particular in that group 8.43: mechanic of motor vehicles and 8.44: mechanics of engines of plane) for 69% of them and 9-7 (group including material-handling and earth-moving machinery drivers, dockers, and freight handlers) and in particular 9.71: dockers, 9.72: riggers, 9.73: driver of overhead crane, and 9.74.60: driver of bitumen and tarring machines for the remaining. Adjustment for asbestos exposure decreases the crude findings, more specifically for the subjects who are classified in the highest class of the diesel CEI, to an extent that depends on the sensitivity or the specificity of asbestos assessment. This result is in coherence with the fact that when restricting the analysis to subjects not exposed to asbestos we find almost no subjects with high diesel exposure. Our group of subjects never exposed to asbestos allowed us to bypass the difficulty to adjust for concomitant exposures and confirm that the relation between diesel fumes exposure and the risk of lung cancer is independent of asbestos exposure. Silica exposure is another carcinogen very frequently found in working places and in particular in places where asbestos or DME is also found (e.g., construction sites). In order to investigate to what extent our results among subjects never exposed to asbestos were not due to silica exposure, we adjusted for this carcinogen and found similar results. Finally, excluding subjects never exposed to asbestos could also modify the distribution of cases and controls according to socioeconomical status. We thus further excluded from this group subjects with high school or university degree and found again a consistent (even if not significant) relationship between DME exposure and lung cancer that was likely not due to silica exposure or asbestos. These results suggest that adjustment for asbestos exposure decreases the association between DME exposure and lung cancer that could explain why the studies that have specifically considered asbestos have in most cases failed to find a significant dose/duration respond relationship.

## 5. Conclusion

Overall, our study highlights DME exposure as a risk factor of lung cancer, a result that is not due to smoking or to asbestos exposure. Recognition of these lung cancers as an occupational disease should be considered. Efforts should be made to reduce DME emissions to decrease the occurrence of lung cancer among the workers exposed to these fumes, even if the composition of DME has evolved over time. Our results highlight that, because DME and asbestos exposure are closely related in the occupational histories of the subjects, analysis by exposure subgroups must be considered. This consideration could also be applicable to other occupational exposures and emphasizes the importance of initiating large-scale studies.

## Figures and Tables

**Table 1:** Selected characteristics of the male study population by case-control status.

	Cases		Controls		OR^1^	95% CI
	Number	%	Number	%		
Total	2264		2780			
Département						
Bas-Rhin	301	13.3	360	12.9		
Calvados	269	11.9	358	12.9		
Doubs + Territoire de Belfort	106	4.7	112	4.0		
Haut-Rhin	56	2.5	89	3.2		
Hérault	251	11.1	360	12.9		
Isère	370	16.3	407	14.6		
Loire Atlantique	269	11.9	311	11.2		
Manche	262	11.6	247	8.9		
Somme	268	11.8	387	13.9		
Vendée	112	4.9	149	5.3		
Age at recruitment, years						
mean (SD)	60 (9.0)		58 (9.9)			
	*p* < 10^−4^					
<50	310	13.7	663	23.8	1.00	[ref]
50–60	767	33.9	855	30.7	1.94	1.64–2.29
60–70	818	36.1	923	33.2	1.89	1.60–2.23
≥70	369	16.3	339	12.2	2.34	1.91–2.86
Highest educational level						
Elementary school or less	672	33.3	521	19.4	1.00	[ref]
Middle school	863	42.8	1081	40.3	0.61	0.53–0.71
High school	185	9.2	310	11.5	0.45	0.36–0.56
University	271	13.4	752	28.0	0.27	0.23–0.33
Unknown	25	1.2	19	0.7	1.05	0.57–1.93
Number of jobs held						
Mean (SD)	4.4 (2.7)		4.6 (2.6)			
	*p = 10* ^−3^					
Cigarette smoking (CSI)						
0	59	2.6	813	29.3	1.0	[ref]
]0–0.5]	105	4.7	613	22.1	2.3	1.6–3.2
]0.5–1]	233	10.4	508	18.3	6.3	4.6–8.5
]1–1.5]	396	17.6	396	14.3	14.7	10.8–19.8
]1.5–2]	768	34.2	333	12.0	34.3	25.4–46.2
CSI > 2	682	30.4	108	3.9	87.7	62.5–122.9
Mean (SD)	1.62 (0.6)		0.65 (0.7)			
	*p* = 10^−4^					
Histological types^2^						
Squamous cell carcinoma	803	35.2				
Adenocarcinoma	794	34.8				
Small cell carcinoma	334	14.6				
Large cell carcinoma	200	8.8				
Other	130	5.7				
Sarcoma	6	0.3				
Nonspecified	13	0.6				

OR: odds ratio CI: confidence interval – CSI: comprehensive smoking index (see Section 2.7).

OR^1^: adjusted for age, department, and CSI (except when CSI is concerned).

^2^16 patients had multiple tumors.

**Table 2:** OR of lung cancer according to previous occupational exposures and to asbestos.

	Cases		Controls		OR^1^	95% CI	OR^2^	95% CI
	Number	%	Number	%				
Total	2264		2780					
Ever worked in List A industries/occupations								
No	1824	80.6	2451	88.2	1.00	[ref]		
Yes	440	19.4	329	11.8	1.78	1.52–2.08		
Cumulative exposure of asbestos (sensitive definition)^3^								
Not exposed	638	28.6	1147	41.5	1.00	[ref]	1.00	[ref]
]0–0.27], low level	613	27.5	808	29.2	1.49	1.25–1.79	1.45	1.21–1.74
]0.27–14], medium level	691	31.0	648	23.5	1.71	1.43–2.05	1.61	1.33–1.94
>14, high level	285	12.8	159	5.8	2.49	1.91–3.25	2.29	1.74–3.02
*Test for trend*, *p*					*<10* ^−4^		*<10* ^−4^	
Cumulative exposure of asbestos (specific definition)^4^								
Not exposed	1189	52.8	1823	65.6	1.00	[ref]	1.00	[ref]
]0–1.63], low level	428	19.0	477	17.2	1.30	1.25–1.79	1.22	1.01–1.47
]1.63–24], medium level	460	20.4	383	13.8	1.53	1.43–2.05	1.41	1.15–1.72
>24, high level	173	7.7	96	3.5	1.88	1.91–3.25	1.70	1.23–2.35
*Test for trend*, *p*					*<10* ^−4^		4 × 10^−4^	

OR: odds ratio; CI: confidence interval; CSI: comprehensive smoking index (see Section 2.7).

1: adjusted for age, département, and CSI.

2: adjusted for age, département, CSI, and List A job.

3: This definition takes into account all job periods, whatever the proportion of workers potentially exposed to asbestos in the job (i.e., sensitive definition) (see Section 2.6).

4: This definition takes into account job periods whose proportion of workers potentially exposed to asbestos is above 30% (i.e., specific definition) (see Section 2.6).

**Table 3:** OR of lung cancer according to diesel motor exhaust (DME) exposure assessed by the questionnaire.

	Cases		Controls		OR^1^	95% CI	OR^2^	95% CI	OR^3^	95% CI	OR^4^	95% CI
	Number	%	Number	%								
Total	2264		2780									
Questionnaires assessment												
Maximum of probability (*P*_*Q*_)												
No exposure	1099	64.6	1641	70.0	1.00	[ref]	1.00	[ref]	1.00	[ref]	1.00	[ref]
Possible exposure	14	0.8	12	0.5	1.39	0.54–3.57	1.28	0.49–3.33	1.08	0.42–2.77	1.16	0.45–3.01
Definite exposure	589	34.6	691	29.5	1.35	1.13–1.61	1.33	1.11–1.58	1.20	1.0–1.44	1.26	1.05–1.51
*Test for trend*, *p*					*0.004*		*0.008*		*0.15*		*0.01*	
Duration of exposure												
0	1099	64.6	1641	69.8	1.00	[ref]	1.00	[ref]	1.00	[ref]	1.00	[ref]
≤15 years	291	17.1	343	14.6	1.38	1.01–1.74	1.33	1.06–1.68	1.22	0.97–1.55	1.27	1.01–1.61
>15 years	312	18.3	367	15.6	1.31	1.05–1.63	1.29	1.04–1.61	1.15	0.92–1.44	1.23	0.98–1.53
*Test for trend*, *p*					*0.03*		*0.03*		*0.26*		*0.09*	
Cumulative exposure index												
0	1099	64.6	1641	69.8	1.00	[ref]	1.00	[ref]	1.00	[ref]	1.00	[ref]
≤31	295	17.3	362	15.4	1.31	1.05–1.65	1.27	1.01–1.60	1.16	0.92–1.47	1.21	0.96–1.53
>31	308	18.1	348	14.8	1.36	1.09–1.70	1.35	1.08–1.68	1.20	0.96–1.51	1.28	1.02–1.60
*Test for trend*, *p*					*0.007*		*0.01*		*0.12*		*0.04*	

OR: odds ratio CI: confidence interval; CSI: comprehensive smoking index (see Section 2.7).

^1^Odds ratio adjusted for age, département, CSI, and number of jobs.

^2^Odds ratio adjusted for age, département, CSI number of jobs, and employment in a List A job.

^3^Odds ratio adjusted for age, département, CSI, number of jobs, and asbestos exposure cumulative index (sensitive definition).

^4^Odds ratio adjusted for age, département, CSI, number of jobs, and asbestos exposure cumulative index (specific definition).

**Table 4:** OR of lung cancer according to diesel motor exhaust (DME) exposure among men not exposed to asbestos.

	Entire Population										Population excluding subjects with high school and university degrees					
	Cases		Controls		OR^0^	95% CI	OR^1^	95% CI	OR^2^	95% CI	Cases		Controls		OR^2^	95% CI
	*N*	%	*N*	%							*N*	%	*N*	%		
Total	638		1147								313		462			
Questionnaire assessment																
Maximum of probability (*P*_*Q*_)																
No exposure	384	77.7	819	83.4	1.00	[ref]	1.00	[ref]	1.00	[ref]	190	72.2	313	76.9	1.00	[ref]
Possible exposure	0	0.0	0	0.0							0	0.0	0	0.0		
Certain exposure	110	22.3	163	16.6	1.46	1.03–2.07	1.47	1.03–2.08	1.44	1.01–2.04	73	27.8	94	23.1	1.31	0.82–2.10
*Test for trend*, *p*					*0.03*		*0.03*		*0.04*						*0.21*	
Duration of exposure																
0	384	77.7	819	83.4	1.00	[ref]	1.00	[ref]	1.00	[ref]	190	72.2	313	76.9	1.00	[ref]
≤15 years	44	8.9	68	6.9	1.36	0.82–2.25	1.36	0.82–2.25	1.30	0.78–2.17	23	8.8	36	8.8	1.13	0.55–2.33
>15 years	66	13.4	96	9.8	1.54	1.00–2.36	1.54	1.00–2.36	1.54	1.00–2.37	50	19.0	58	14.3	1.42	0.82–2.47
*Test for trend*, *p*					*0.047*		*0.047*		*0.047*						*0.21*	
Cumulative exposure index																
0	384	77.7	819	83.3	1.0	[ref]	1.00	[ref]	1.00	[ref]	190	0.70	313	0.77	1.00	[ref]
<median (]0–31])	44	8.9	72	7.3	1.27	0.77–2.09	1.27	0.77–2.10	1.22	0.74–2.01	23	0.09	37	0.09	1.07	0.52–2.20
>median (>31)	66	13.4	92	9.4	1.62	1.05–2.51	1.63	1.05–2.52	1.62	1.05–2.52	50	0.19	57	0.14	1.47	0.84–2.55
*Test for trend*, *p*					*0.03*		*0.03*		*0.03*						*0.17*	

OR: odds ratio CI: confidence interval; CSI: comprehensive smoking index (see Section 2.7).

OR^0^: adjusted for age, département, CSI, and number of jobs.

OR^1^: adjusted for age, département, CSI, number of jobs, and employment in a List A job.

OR^2^: adjusted for age, département, CSI, number of jobs, and cumulative exposure index of silica exposure.
